# The Effect of Compression Pressure on the First Layer Surface Roughness and Delamination of Metformin and Evogliptin Bilayer and Trilayer Tablets

**DOI:** 10.3390/ph16111523

**Published:** 2023-10-26

**Authors:** Sun Ho Kim, Jung Han Kook, Dong-Wan Seo, Myung Joo Kang

**Affiliations:** 1 College of Pharmacy, Dankook University, 119, Dandae-ro, Dongnam-gu, Cheonan-si 31116, Republic of Korea; cooloveu7@naver.com; 2 KS TECH, 1223-24, Cheonan-daero, Seobuk-gu, Cheonan-si 31080, Republic of Korea; key.kstech@gmail.com

**Keywords:** multi-layer tablet, delamination, surface roughness, energy dispersive spectrometry analysis, inter-penetration of particles, profilometer, interfacial strength

## Abstract

The objectives of this study were to evaluate the delamination of convex-shaped metformin HCl (MF) and evogliptin tartrate (EG) multi-layer tablets depending on the pre-compression and main compression pressures and simultaneously correlate these results with those of a surface roughness analysis. Free-flowing MF and EG (median diameters of 38.3 and 44.7 μm, respectively) granules prepared using the wet granulation method were pre-compressed and subsequently compressed into bilayer and trilayer tablets using a universal testing machine. The compaction force required to break the tablets increased linearly as the main compression pressure increased (30–150 MPa). Conversely, the interfacial strength and compaction breaking force decreased as the pre-compression pressure increased (10–110 MPa). A surface roughness analysis employing a profilometer revealed that the first layer (MF) roughness drastically decreased from 5.89 to 0.51 μm (Ra, arithmetic average of profile height deviations from the mean line) as the pre-compression pressure increased from 10 to 150 MPa in the bilayer tablet. Accordingly, the decrease in the roughness of the first layer reduced the inter-penetration at the interface, as observed via energy dispersive spectrometer (EDS)-equipped scanning electron microscopy, decreasing the interfacial bonding strength and causing delamination of the MF/EG multi-layer tablets. These findings indicate the significance of roughness control in the actual preparation of multi-layer tablets and the usefulness of profilometer- and EDS-based surface analyses for interpreting the delamination of multi-layer tablets.

## 1. Introduction

Multi-layer tablets (MLTs), including bilayer or trilayer tablets, are gaining considerable attention as effective tools for fixed-dose combination (FDC) therapy, with advantages over conventional monolithic tablets [1,2]. Incompatible active ingredients can be formulated in separate layers, minimizing the contact area and providing a better physicochemical stability [3]. Moreover, MLTs can be designed to simultaneously provide immediate- and slow-release layers to provide the desired drug release profile for individual ingredients in a single-dosage form [4,5,6]. However, despite the several advantages of the MLT system, the fabrication process is complicated, with steps including die filling for the first layer, pre-compression, die filling for the second layer, main compression, unloading, and ejection [7]. This complex process occasionally causes more technical challenges compared to conventional monolithic tablets, including tablet defects such as delamination separation of individual layers at the interfaces during manufacturing, shipping, and storage [8,9].

Various physical and mechanical studies have been conducted to understand the factors that contribute to delamination or cracks in MLTs. Tablet defects are principally associated with interfacial adhesion between adjacent layers and the mechanical integrity of the solid dosage form [10]. Moreover, the difference in deformation and/or elastic recovery between the adjacent layers contributes to radial stress, causing delamination of the MLTs [11,12]. The interfacial bonding strength is reportedly significantly influenced by the compression properties of the individual layers and the process parameters, particularly the compression pressure and punch speed [12]. An appropriate compression pressure on the first layer (pre-compression) is required to flatten the first layer surface, reduce the volume of powder/granulated substances, and provide space to place the second layer [13,14]. However, the application of excess compression pressure leads to a lower interfacial roughness, which could promote MLT delamination by diminishing the intermolecular adherence between adjacent layers [15,16]. Particularly, when a plastic material (e.g., methylcellulose) is included in both layers, an increased pre-compression pressure (PRE-P) applied to the first layer causes a decline in the interfacial strength of the bilayer tablets [17]. To date, most mechanistic studies have been conducted using one or two components made of common pharmaceutical excipients, such as microcrystalline cellulose, lactose, or starch. However, the compression of one or two components does not represent actual pharmaceutical formulations, especially granules fabricated using wet or dry granulation methods, which are composed of drug substances, diluents, disintegrants, binders, and lubricants. Furthermore, to the best of our knowledge, quantitative measurements of surface roughness and its relationship with the mechanical strength of bilayer and trilayer tablets have not yet been reported to date.

Therefore, we aimed to evaluate the effect of the compression pressure on the surface roughness of first layer, the compaction force required to break the MLTs, and the interfacial strength, and their correlations in the MLT pharmaceutical product. As a model product, metformin HCl (MF) and evogliptin tartrate (EG) fixed-dose combination tablets, currently prescribed to treat type 2 diabetes mellitus, were employed [18,19,20]. MLTs are oval-shaped, convex tablets consisting of a sustained-release (SR) MF layer and an immediate-release (IR) EG layer [21]. The exact compositions of the mixtures subjected to granulation processes are listed in Table 1. In this study, the compaction force required to break MF/EG MLTs and the interfacial strength between the adjacent MF and EG layers depending on different PRE-Ps and main compression pressures (MAIN-Ps) were determined. Subsequently, the surface roughness of the first layer was quantitatively analyzed using a profilometer, and the correlation between the compaction breaking force and interfacial strength was investigated. A profilometer was used to determine commonly applied roughness parameters, and attempts have been made to correlate these values with other characteristic material parameters. Furthermore, the inter-penetration of the active ingredient of the first layer (MF) into the second layer (EG), depending on the PRE-P, was analyzed using energy dispersive spectrometer (EDS)-equipped scanning electron microscopy (SEM).

## 2. Results and Discussion

### 2.1. Morphological and Physical Characteristics of MF and EG Granules

In this study, both EG- and MF-loaded granules were prepared using wet granulation, and their morphology, particle size, density, and flowability were evaluated. The efficiencies (%) of the granulation processes for EG and MF were determined to be 98.7% and 99.8%, respectively. For MF granules, the drug ratio was designed to be approximately 80%, considering the large clinical dose of MF (500–1000 mg/day) [22]. Carbomer 934P, hypromellose (hydroxymethyl cellulose, HPMC2208), and methacrylate copolymer were included in the MF-loaded granules for the sustained release of MF, deaccelerating the rapid disappearance of the drug in plasma after oral administration [23]. Conversely, mannitol-based granules were designed for EG delivery, ensuring rapid disintegration and release. Ac-di-sol and low-substituted hydroxypropyl cellulose (L-HPC) were included as disintegrants in the EG-loaded granules to promote their disintegration and subsequent deaggregation (Table 1).

**Figure 1:**
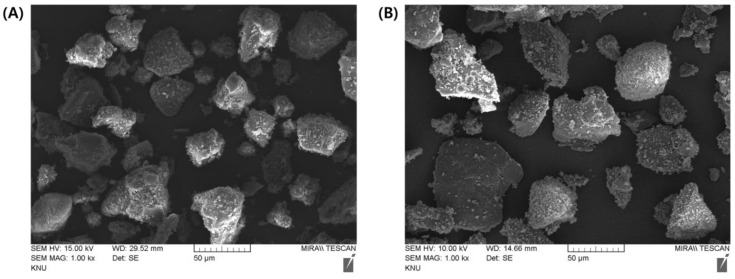
Representative FE-SEM images of the (**A**) MF and (**B**) EG granules. The samples were coated with platinum and analyzed at 15 and 10 kV, respectively. (**A**,**B**) are ×1000 magnified images. Abbreviations: EG, evogliptin tartrate; MF, metformin HCl; SEM, scanning electron microscopy.

SEM observations revealed that spherical and fibrous granules were present in a mixed form in both the EG- and MF-loaded granules. The granular size was 20–100 μm in both granules, with rough and irregular surfaces (Figure 1). The particle size determined using a particle size analyzer coincided with that observed in SEM; the median particle size (d_0.5_) of the EG granule was measured to 38.33 μm, with a homogeneous size distribution (SPAN value, 1.76) (Table 2). In contrast, the particle size of the MF-loaded granules was 44.74 μm (SPAN value, 2.24). The losses on drying (LODs) of EG granules and MF granules were determined to be 0.70% and 0.74% *w*/*w*, respectively, denoting that both granules were appropriately desiccated by drying (Table 2). The bulk density (BD) and tapped density (TD) of the EG granule were determined to 0.46 and 0.51 g/mL, respectively. Moreover, the BD and TD of the MF granule were 0.43 and 0.48 g/mL, respectively. The Carr’s index (CI) and Hausner’s ratio (HR) were calculated using the BD and the TD as indicators of flowability of the granules (Table 2). A CI of >25 is considered an indicator of poor flowability, while values *<* 15 indicate good flowability [24]. Similarly, a lower HR of the granules indicates better flowability. A HR of <1.11 is regarded as an “excellent” flow, whereas a HR of >1.60 indicates “very poor” flowability. There are intermediate scales for CI between 11 and 15 or HR between 1.12 and 1.18 (Table 2), which indicates “good” flowability. The CI and HR values of EG-loaded granules were calculated to be 9.8% and 1.11 (Table 2), suggesting that EG-loaded granules have “excellent” flowability. On the other hand, these values in MF-loaded granules were determined to be 10.4% and 1.12 (Table 2), respectively, and are considered to indicate an “excellent” or “good” flowability. Then, the free-flowing granules were then employed to fabricate MF/EG bilayer and trilayer tablets to evaluate the effects of the PRE-P and MAIN-P on the compaction breaking force and interfacial strength of the tablets.

### 2.2. Effect of Compression Pressure on the Porosity and Compaction Breaking Force of MF/EG MLTs

**Figure 2:**
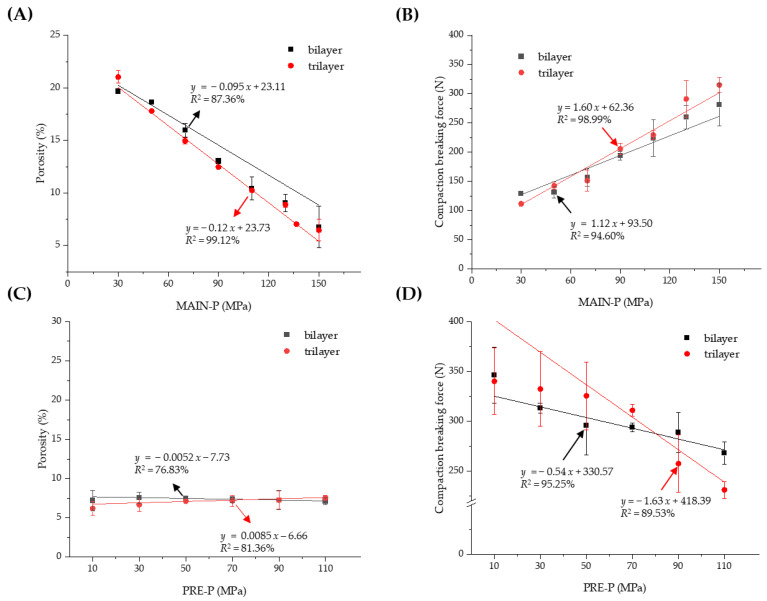
Effect of the MAIN-P and PRE-P on the porosity and compaction breaking force of MF/EG MLTs. Porosity (**A**) and compaction breaking force (**B**) of MLT with different MAIN-Ps, and porosity (**C**) and compaction breaking force (**D**) of MLT prepared with different PRE-Ps. Notes: In (**A**,**B**), the PRE-P was fixed at 50 MPa and the MAIN-P was varied from 30 MPa to 150 MPa during compression. In (**C**,**D**), the PRE-P was varied from 10 MPa to 110 MPa and the MAIN-P was fixed at 150 MPa. The trilayer tablet was pre-compressed twice with identical compression pressures. Abbreviations: EG, evogliptin tartrate; MAIN-P, main compression pressure; MF, metformin HCl; MLT, multi-layer tablet; PRE-P, pre-compression pressure.

The granules were compressed using a universal testing machine (JP/AG-50kNX, Shimadzu, Kyoto, Japan) to prepare the MF/EG MLTs. Compression using a universal testing machine provides advantages such as precise control over the compression force and speed, real-time data collection, and understanding of material behavior [25]. The porosity and compaction breaking force of MF/EG bilayer and trilayer tablets depending on PRE-Ps and MAIN-Ps are represented in Figure 2. At first, the porosity of the MF/EG bilayer and trilayer tablets was measured with different MAIN-Ps ranging from 30 to 150 MPa, with the PRE-P fixed at 50 MPa (Figure 2A). As expected, the porosity of the tablets decreased linearly as the MAIN-P increased, and the porosities of the bilayer and trilayer tablets were determined to be 6.75% and 6.47%, respectively, at 150 MPa, which are approximately one-third of those obtained at 30 MPa. Increasing the compression pressure may promote the fragmentation, deformation, and densification of granules, thereby decreasing the intragranular pores and voids during compression. Accordingly, increasing the MAIN-P strengthened the mechanical integrity of the MF/EG bilayer and trilayer tablets. When the MAIN-P increased from 30 to 70, 110, and 150 MPa, the compaction force required to delaminate the bilayer tablet increased by 129, 157, 224, and 281 N, respectively (Figure 2B). The trilayer tablets also exhibited a similar pattern; the force to delaminate the trilayer tablets increased to 111, 151, 229, and 315 N, when the compression increased from 30 to 70, 110, and 150 MPa, respectively. When the compaction breaking force was plotted against the MAIN-P, a linear relationship was observed for bilayer and trilayer tablets (R^2^ = 0.9 for both types).

Next, the porosity and compaction breaking force of the MF/EG bilayer and trilayer tablets depending on the PRE-P were evaluated using a fixed MAIN-P value (150 MPa). As shown in Figure 2C, despite the increase in the PRE-P from 10 to 110 MPa, the porosity of the bilayer tablet ranged from 7.12% to 7.54%. Similarly, the porosity of the trilayer tablet was adjusted to a range of 6.19% to 7.55%, because a higher MAIN-P value (150 MPa) than the PRE-P predominantly influences the thickness and porosity of the tablets. Interestingly, despite the comparable porosity of the matrices, the increase in the PRE-P caused a linear decrease in the force required to cause breakage or delamination of the MF and EG bilayers (Figure 2D). The bilayer tablet compressed with a 10 MPa PRE-P exhibited a compaction breaking force of 346 N, whereas that of the tablet pre-compressed at 110 MPa was estimated to be 268 N. This pattern was further intensified in the trilayer tablet; when the PRE-P increased from 10 to 110 MPa, the force required for breakage or delamination decreased from 340 to 231 N.

### 2.3. Effect of PRE-P on the Interfacial Strength of MF/EG MLTs

**Figure 3:**
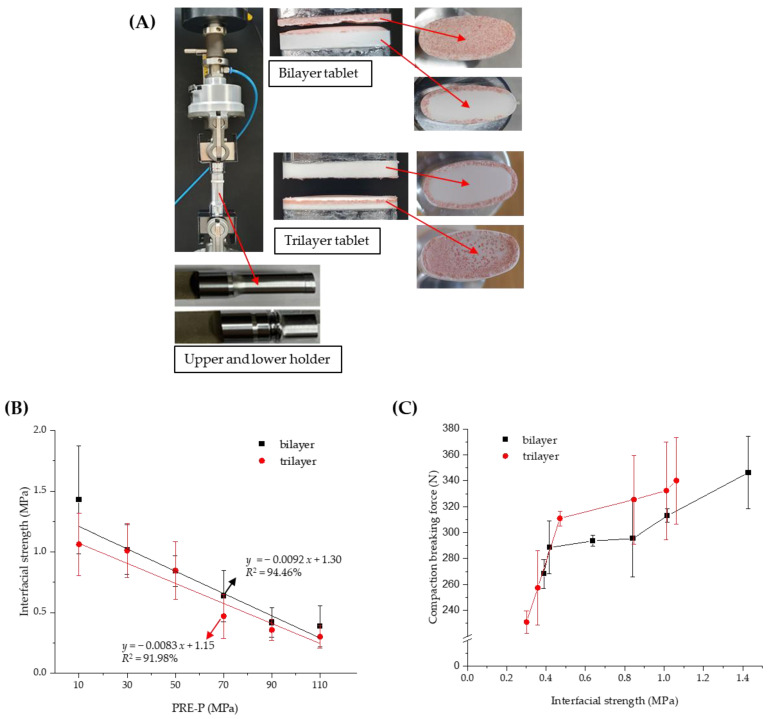
Effect of the PRE-P on the interfacial strength of MF/EG MLTs. (**A**) Image of the interfacial strength tester with a newly designed holder for convex-shaped tablets. (**B**) Interfacial strength of MF/EG MLTs with different PRE-Ps. (**C**) Relation between compaction breaking force and interfacial strength in MF/EG MLTs, varying the PRE-P. Notes: the PRE-P was varied from 10 to 110 MPa, and the MAIN-P was fixed at 150 MPa. The trilayer tablet was pre-compressed twice at identical pressures. Abbreviations: EG, evogliptin tartrate; MF, metformin HCl; MLT, multi-layer tablet; PRE-P, pre-compression pressure.

Several methods have been developed to quantify the strength of bilayer tablets, such as the tensile, shear, and diametrical compression; three-point bending; and V-shaped punch-breaking tests [26,27,28,29]. The interfacial strength was further determined to estimate the strength of the MF/EG bilayer and trilayer tablets along with the compaction breaking force, as described in Section 2.2. Interfacial tensile tests are currently recommended for measuring the interfacial bonding strength of MLTs because stress is more uniformly applied in a direction perpendicular to the interface and is not affected by the tablet direction [30]. To determine the interfacial strengths of the MF/EG bilayer and trilayer tablets, an interfacial strength tester equipped with a newly designed holder suitable for convex-shaped tablets was used (Figure 3A). Although conventional equipment is used to determine the interfacial strength of flat tablets, it is challenging to measure the interfacial strength of convex tablets using this equipment. Figure 3A shows the delamination of the bilayer and trilayer tablets during the experiment. Both the bilayer and trilayer tablets were split at the interface. In the case of trilayer tablets, delamination mostly occurred between the first layer (MF) and the second layer (EG), but occasionally between the second layer (EG) and the third layer (MF).

As shown in Figure 3B, the interfacial strength of MF/EG MLTs tended to linearly decrease with an increasing PRE-P in both the bilayer and trilayer tablets, similar to the compaction breaking force. Moreover, the interfacial strength of the bilayer tablet decreased from 1.43 to 0.39 MPa as the PRE-P increased from 10 to 110 MPa (Figure 3B). The interfacial strength of the trilayer tablet also decreased from 1.06 to 0.30 MPa as the PRE-P increased from 10 to 110 MPa. Thus, the trends in compaction breaking force and interfacial strength were correlated with an R^2^ value of 0.93 for the bilayer tablet (Figure 3C).

### 2.4. Effect of Pre-Compression Pressure on the Surface Roughness of MF/EG Bilayer Tablets

Previous studies have revealed that the physicochemical properties of powders and the manufacturing conditions affect the interfacial strength of MLTs. Kottala et al. [31] produced bilayer tablets using plastic and brittle materials in different layers and determined the interfacial adhesion strength of the layered tablets using a direct tensile test. They revealed that the difference in material elasticity between the layers should be minimized to increase tablet integrity [12]. Zhang et al. (2018) [32] reported that the particle size in the first layer, the powder water, and the punch shape used for pre-compression affected the interfacial strength. The first layer made from a coarse powder (i.e., a large particle size) had a rougher interfacial surface, promoting particle interactions with adjacent layers. Based on these findings, the roughness of the tablet interface depending on the PRE-P was considered as the main indicator for interpreting the delamination of the MF/EG bilayer and trilayer tablets.

Several factors have been proposed to explain lamination, including high compression speeds [33], high die-wall residual stresses [34], anisotropic mechanical properties [35], air entrapment [36], and the surface roughness of the pre-compressed layer [15]. In this study, in order to establish a correlation between surface roughness and the interfacial strength required for tablet delamination of MF/EG multi-layer tablets, we employed the commercial euro standard punch, which is employed in the actual production of MF/EG multi-layer tablets. Accordingly, we expect that the result obtained with the universal testing machine could be applicable when compacting using instrumented standard compaction equipment.

**Figure 4:**
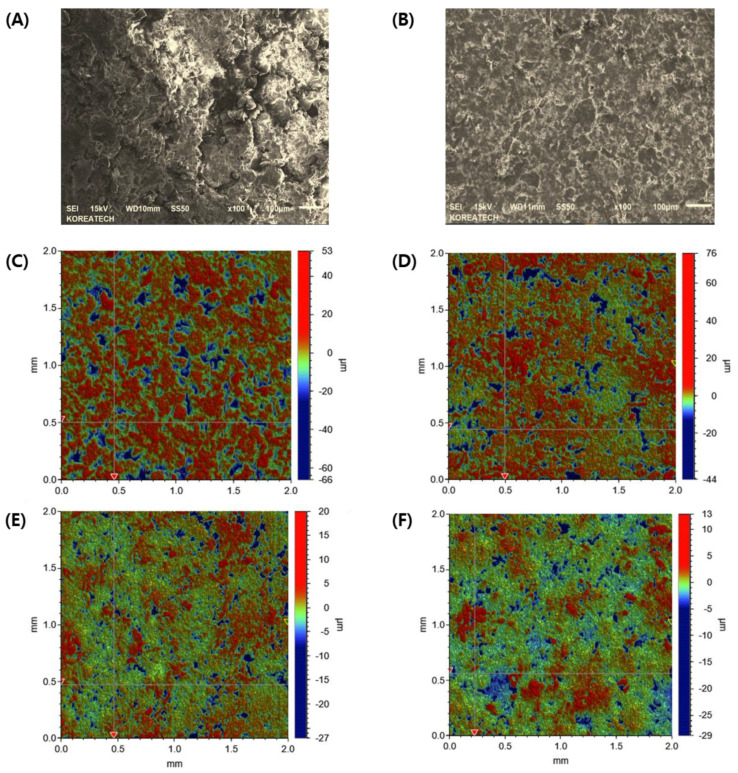
Morphological observations and quantitative analysis of the surface topography of the first layer compressed using different PRE-Ps with no main compression process. Representative FE-SEM images of the first layers pre-compressed using pressures of (**A**) 30 MPa and (**B**) 150 MPa. Quantitative analysis of the surface roughness of the first layers compressed with different PRE-Ps of (**C**) 30 MPa, (**D**) 50 MPa, (**E**) 70 MPa, and (**F**) 150 MPa. Note: To analyze the surface topography of the first layers, a stylus profilometer was employed with a scanning rate and size of 285 µm/s and 2500 µm. The red-colored dots indicate a roughness over 4 µm, while the green-colored dots indicate a roughness in the range of 0.5–1 µm. Abbreviations: PRE-P, pre-compression pressure; SEM, scanning electron microscopy.

**Figure 5:**
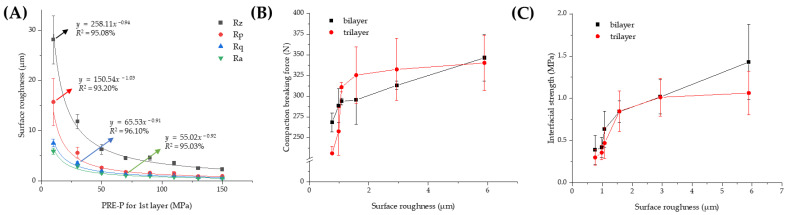
Effect of the PRE-P on the roughness (Ra) of the first layer and its correlation with the interfacial strength or compaction breaking force. (**A**) Profile of the surface roughness of the first layers prepared using different PRE-Ps with no additional compression process. Correlation of surface roughness with (**B**) compaction breaking force and (**C**) interfacial strength of MF/EG MLTs. Note: a stylus profilometer was employed to analyze the surface topography of the first layers, with a scanning rate and size of 285 µm/s and 2500 µm. Abbreviations: EG, evogliptin tartrate; MF, metformin HCl; MLT, multi-layer tablet; PRE-P, pre-compression pressure.

At first, the interfacial topography and roughness of the first layer pre-compressed at different pressures with no main compression process were observed using FE-SEM. In the first layer pre-compressed with a 30 MPa pressure, drug-loaded granules were not completely fragmented, and the shape of the granules was preserved with a rough surface (Figure 4A). Conversely, when pre-compressed with a higher PRE-P (150 MPa), the shape of each granule was not observed, indicating that each granule might have been fragmented and deformed by the strong pressure and that the granules interconnected to form a flat surface (Figure 4B). The interfacial topography of the first layer, pre-compressed at different pressures, was further scrutinized using a stylus profilometer. The stylus profilometer uses a contact-based technique to analyze surface topography using a probe that moves physically along the surface to acquire surface characteristics such as height. It provides the advantage of long-distance measurements and a clear wave profile of the surface roughness with high precision [37]. Figure 4C–F shows representative profilometer images of the individual first layers pre-compressed with different PRE-Ps (30, 50, 70, and 150 MPa). When the first layer was compressed with 30 MPa, many parts expressed in a red color with a roughness of >4 μm were prevalent. Conversely, as the PRE-P increased, the roughness of the first layer decreased remarkably, and when the pressure was >70 MPa, green-colored dots, indicating a roughness between 0.5 and 1 μm were predominant, with no red-colored dots. The changes in the roughness parameters, including Rz (maximum height of the profile), Rp (maximum profile peak height), Rq (root mean square roughness), and Ra (average roughness), with different PRE-Ps are shown in Figure 5A. As the PRE-P increased from 10 MPa to 50 MPa, Rz, Rp, Rq, and Ra decreased rapidly. The values of Rz, Rp, Rq, and Ra under a PRE-P of 10 MPa were measured as 28.09, 15.72, 7.53, and 5.89 μm, respectively, whereas the values of Rz, Rp, Rq, and Ra at 50 MPa were 6.25, 2.62, 1.91, and 1.57 μm, respectively. Then, as the PRE-P increased to 150 MPa, all the roughness parameters gradually decreased, resulting in Rz, Rp, Rq, and Ra values of 2.28, 0.95, 0.63, and 0.51 μm, respectively.

After quantitative analysis of the roughness of the first layers depending on the PRE-P, its relationship with the mechanical strength of the MLTs, including the compaction breaking force or interfacial strength, was evaluated. As a parameter for interfacial roughness, Ra (arithmetic average of profile height deviations from the mean line), a representative parameter for surface roughness, was employed [38]. As shown in Figure 5B, as the surface roughness increased, the compaction force required to delaminate the tablets increased. When the surface roughness reached 2 μm, the compaction breaking force was steeply amplified as the roughness increased. Then, a moderate increase was observed for both the bilayer and trilayer tablets. The interfacial strength also exhibited an analogous profile (Figure 5C); it increased steeply as the roughness increased to 2 μm, followed by a steady increase at a roughness of 6 μm. For the bilayer tablet, when the Ra values were 0.76, 1.57, 2.94, and 5.89 μm, the interfacial strengths were determined to be 0.39, 0.84, 1.02, and 1.43 MPa, respectively. In the case of the trilayer tablets, the interfacial strength was measured as 0.30, 0.85, 1.01, and 1.06 MPa when the Ra values were increased to 0.76, 1.57, 2.94, and 5.89 μm. As the surface roughness of the first layer decreased, the void volume was reduced in both within the powder bed and at the surface, thus causing a limited penetration of particles from the first layer into the second layer. Accordingly, bonding between the two layers is confined to the interface, with little interdigitation between the layers [39]. Moreover, the contact area for the second layer was significantly reduced at the interface, resulting in a weaker adhesion of the adjacent layers at the interface. These results were consistent with those of previous reports, stating that a certain amount of interfacial roughness in the initial layer is essential for particle interlocking and adhesion to the adjacent layer [40]. Desai et al. (2013) [41] also reported that smoothing the surface of the first layer caused delamination of the MLT by limiting the intermolecular adherence between adjacent layers.

### 2.5. EDS-Equipped SEM Observation of MF/EG Bilayer Tablets

The migration of the active ingredient of the first layer into the second layer depending on the PRE-P was further analyzed using EDS-equipped SEM observations. Chang et al. [15] revealed that the extent of inter-penetration depends on both the material properties and compression pressure, which affect the surface waviness and porosity at the interface. In this study, in order to scrutinize the migration of the inter-penetration of the MF-containing first layer into the EG-containing second layer, the distribution of MF at the interface of the bilayer tablet was analyzed. To track the MF, chloride (Cl), an element included only in the MF molecule, was tracked using EDS analysis.

**Figure 6:**
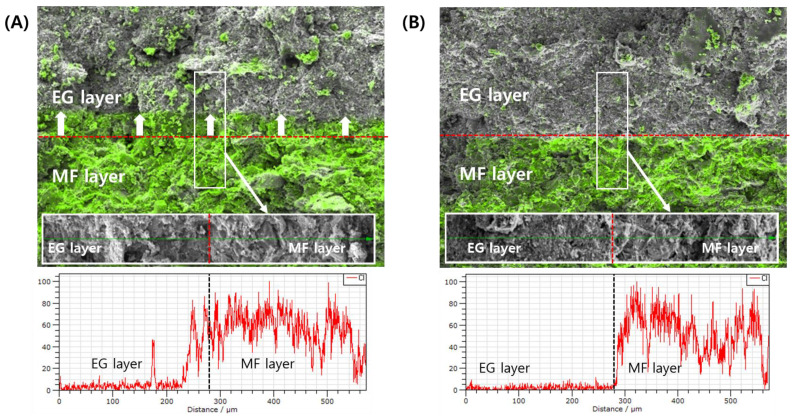
EDS-equipped FE-SEM analyses of the inter-penetration of MF powder at the interface of EG/MF bilayer tablets during the compression process. (**A**) Analysis of MF/EG bilayer tablets prepared with PRE-P and MAIN-P pressures of 10 MPa and 150 MPa, respectively. (**B**) Analysis of MF/EG bilayer tablets prepared with PRE-P and MAIN-P pressure of 110 MPa and 150 MPa, respectively. Notes: EDS-equipped FE-SEM analyses were performed on cross-sectioned bilayer tablets, after pre-compression and subsequent main compression processes. The distribution of chloride (Cl) in tablets, an element included in the MF molecule, was tracked using an EDS. The Cl element distributed in the tablets was expressed with a fluorescent green color. The red dotted line represents the interface of the bilayer tablets. White arrows in (**A**) indicate the direction of inter-penetration of MF particles during the compression process. Abbreviations: EDS, energy dispersive spectrometer; EG, evogliptin tartrate; MAIN-P, main compression pressure; MF, metformin HCl; MLT, multi-layer tablet; PRE-P, pre-compression pressure; SEM, scanning electron microscopy.

EDS analysis revealed that in tablets compressed with a low PRE-P (PRE-P and MAIN-P values of 10 and 150 MPa, respectively), the MF contained in the first layer actively interpenetrated the EG-loaded second layer (Figure 6A). MF, expressed with a fluorescent green color, was confirmed in the EG second layer adjacent to the interface, and MF was even detected at a point approximately 100 μm away from the interface. Conversely, in the tablets compressed with a PRE-P of 110 MPa, minimal migration of MF into the second layer was observed (Figure 6B). As the PRE-P increased, the surface of the first layer became denser and smoother, decreasing the inter-particulate attraction and mechanical interlocking between the two adjacent layers. This coincided with a previous report stating that the surface roughness of Avicel in the first layer was reduced significantly with increasing compression pressure (0.5, 1, and 2 kN), causing a decrease in inter-particulate attraction and mechanical interlocking [17]. Our findings suggest that the roughness of the first layer, depending on the PRE-P, drastically affects the delamination problem in bilayer and trilayer tablets prepared by the wet granulation method, and profilometer and EDS analyses can be efficient tools for understanding the interfacial bonding strength and delamination of MLTs.

## 3. Materials and Methods

### 3.1. Materials

MF (median diameter of 38.33 μm) and EG drug powders (median diameter of 44.74 μm) were obtained from Granules India Limited (Madhapur, Hyderabad, India) and Dong-A ST (Seoul, Republic of Korea), respectively. Polyvinylpyrrolidone (PVP K30) was purchased from BASF (Ludwigshafen Land, Rheinland, Pfalz, Germany). High-viscosity grade HPMC2208 (Methocel K100M) and methacrylic acid copolymers (Eudragit S100) were obtained from Dow Chemical (Montgomeryville, PA, USA) and Evonik (Essen, NRW, Germany), respectively. Carbomer 934P (Carbopol^®^ 934P-NF) and mannitol (Pearlitol 100 SD) were obtained from BF-Goodrich (Cleveland, OH, USA) and Roquette (Lestrem, Pas de Calais, France), respectively. Low-substituted hydroxypropyl cellulose (L-HPC) and hydroxypropyl cellulose (Klucel LF) were obtained from Shin-Etsu Chemical (Otemachi, Chiyoda-ku, Japan) and Ashland (Wilmington, DE, USA), respectively. Pregelatinized starch (Starch 1500) and croscarmellose sodium (Ac-Di-Sol) were supplied by Colorcon (Harleysville, PA, USA) and FMC Corp. (Philadelphia, PA, USA), respectively. Colloidal silicon dioxide (Aeroperl 300) was purchased from Evonik (Essen, Germany). Magnesium stearate and red iron oxide (color index number of 77491) were acquired from FACI Asia Pacific (Merlimau Pl, Jurong Island, Singapore) and Univar (Billericay, Essex, UK), respectively.

### 3.2. Preparation of MF and EG Granules Using the Wet Granulation Method

Both MF- and EG-loaded granules were prepared using wet granulation for a batch of 300,000 tablets [42]. The exact compositions of the mixtures subjected to granulation processes are listed in Table 1. To prepare the MF-loaded granules, Eudragit S100 and PVP K30 were first dissolved in ethanol and purified water (4:1 *w*/*w*) as the binder solution using an impeller mixer (SH-HM-3S, Samhung Tech, Incheon, Republic of Korea). The solution was sprayed through a nozzle onto the MF drug powder in a fluid bed granulator (FIF200, Freund, Tokyo, Japan) to form granules. The parameters of the granulation process were as follows: inlet air temperature of 70 °C, airflow rate of 2/4 m^3^/h, exhaust temperature of 37–40 °C, and binder solution feeding rate of 500 mL/min. Subsequently, the dried granules were sieved using a 1.2 mm mesh and admixed with HPMC2208 and Carbopol^®^ 934P in a container mixer (Servolift GmbHd, Offenburg, Germany) at 10 rpm for 10 min. Magnesium stearate, sieved through a 40-mesh sieve, was added to the mixture and lubricated at 10 rpm for 5 min.

EG-loaded granules were prepared using a high-speed mixer (SM-100, Sejong Pharmatech, Incheon, Republic of Korea). The drug powder, mannitol, pregelatinized starch, L-HPC, and colloidal silicon dioxide were added to the chamber and mixed for 5 min with an agitator and chopper at speeds of 75 and 1500 rpm, respectively. Then, an aqueous binder solution containing HPC and iron oxide at concentrations of 0.075 and 0.01 mg/mL, respectively, was sprayed into the mixture for 5 min at agitator and chopper speeds of 75 and 1500 rpm, respectively. The wet granules were dried using a fluid bed granulator (NJ-FBD0200, Namjoo Machinery, Hwaseong, Republic of Korea) for 25 min at an inlet air temperature and an exhaust temperature of 50 and 35 °C, respectively. The dissipated granules were sieved through a 0.8 mm sieve using an oscillator (OscilloWitt, Frewitt, Switzerland). Croscarmellose sodium was further added to the dried granules as a disintegrant in a container mixer (Servolift GmbHd, Offenburg, Germany) and mixed at 15 rpm for 10 min. Magnesium stearate, sieved through a 40-mesh sieve, was added to the mixture at 15 rpm for 3 min.

### 3.3. Characterization of MF and EG Granules Prepared by the Wet Granulation Method

The prepared drug-loaded granules were characterized in terms of drying (LOD), morphology, particle size, density, and flowability (CI and HR). The LOD (%) of the granules (approximately 5 g) was determined using a halogen moisture analyzer (HR73, Mettler Toledo, Leicester, UK) at 105 °C for 15 min until an equilibrium state was reached [43]. For morphological observations, each granule was loaded onto a copper stub using double-sided carbon tape (Sungho Sigma, Suwon, Republic of Korea) and coated with a thin platinum layer using an automatic sputter coater (Model 108AUTO, Cressington Scientific Instruments, Cressington, UK). Microphotographs of the coated samples were obtained using SEM (MIRA3 LMH, TESCAN, Brno, Czech Republic) at acceleration voltages of 10 and 15 kV. The particle size of the granules was analyzed using a particle size analyzer (SALD-2300, Shimadzu Co., Kyoto, Japan) under an air pressure of 0.5 bar using a dry measurement method. As an indicator of the homogeneity of the size distribution, the SPAN value was calculated using the following equation: SPAN = (d_0.9_–d_0.1_)/d_0.5_, where d_0.1_, d_0.5_, and d_0.9_ represent the particle sizes below which 10%, 50%, and 90% of the sample particles lie, respectively [44]. The BD and TD of the drug-loaded granules were determined according to the United States Pharmacopeia (USP) 43, General Chapter 616 [45]. Granules (100 g) were poured into a 250 mL graduated cylinder, and the BD was calculated as the mass divided by the apparent volume of the granules in the cylinder. The granule-loaded cylinder was put into a TD tester (BeDensi T3, Bettersize, Dandong, China) and was tapped 1250 times. The TD (g/mL) was calculated as the weight divided by the volume of the tapped powder. The flowability and compressibility of the granules were estimated by calculating the CI and HR. The CI was calculated from the BD and TD using the following equation: (1 − BD/TD) × 100%. The HR was calculated by dividing the TD by the BD.

### 3.4. Preparation of MF/EG-Loaded Bilayer and Trilayer Tablets

**Figure 7:**
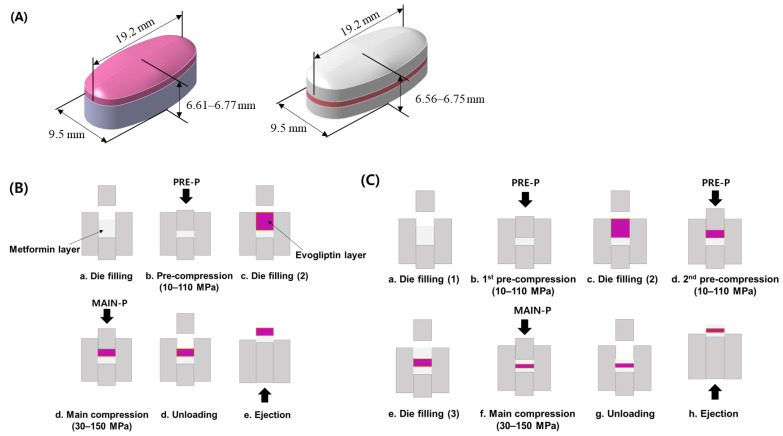
Schematic illustration of MF/EG MLTs and the fabrication process. (**A**) MF/EG bilayer tablet (left) and trilayer tablet (right). Fabrication processes of (**B**) MF/EG bilayer tablets and (**C**) trilayer tablets. Abbreviations: EG, evogliptin tartrate; MAIN-P, main compression pressure; MF, metformin HCl; MLT, multi-layer tablet; PRE-P, pre-compression pressure.

MF/EG-loaded bilayer and trilayer tablets were fabricated by sequential compression of EG- and MF-loaded granules, as depicted in Figure 7 [42]. For the preparation of bilayer tablets, the MF-loaded granules were manually filled into a die. The granules were pre-compressed using a universal testing machine (JP/AG-50kNX, Shimadzu, Kyoto, Japan) at different PRE-Ps (10–110 MPa) to prepare the first tablet layer. The testing machine was equipped with a commercial euro standard D441 tool punch and an oval-shaped, convex-type die with a long axis, a short axis, and a thickness of 19.2 mm, 9.5 mm, and 6.61–6.77 mm, respectively. Then, the EG-loaded granules were added to the first layer (MF layer) and compressed at a MAIN-P between 30 and 150 MPa. The MF layers were colored white, whereas the EG layers were colored red. Then, the tablets were ejected from the die by pushing the first layer upwards with a punch. The compression and ejection speeds were set to 50 and 800 mm/min, respectively.

In the case of the trilayer tablets, MF-loaded granules were manually filled into the die and pre-compressed at different PRE-Ps (10–110 MPa) to form the first layer. Then, the EG granules were manually filled in the first layer and were subsequently pre-compressed with the same pressure as the first PRE-P. Finally, the MF-loaded granules were added to the bilayer and subsequently compressed at compression pressures of 30–150 MPa. The thicknesses of the trilayer tablets ranged between 6.56 and 6.75 mm.

### 3.5. Porosity of MF/EG-Loaded MLTs Depending on Compression Pressure

To determine the porosity (*ε*) of MLTs compressed with different PRE-Ps and MAIN-Ps, the true density was measured by measuring the true volume of the sample using a helium Ultrapyc 1220e Automatic Gas Pycnometer (Anton Paar QuantaTec Inc., Boynton Beach, FL, USA). Then, the porosity (*ε*) was calculated using the following the equation:

(1) $$\varepsilon =(1-\frac{m}{\rho_{t}v})\times 100$$

where *ρ_t_* is the true density, *m* is the weight of the tablet, and *v* is the volume of the tablet. The apparent volume of the tablet was calculated using a 3D modeling program (CATIA V5R21, Dassault Systemes, Vélizy-Villacoublay, France).

### 3.6. Compaction force Required to Break MF/EG-Loaded MLTs

The compaction force required to crush or delaminate MF/EG-loaded MLTs was determined using a universal testing machine (JP/AG-50kNX; Shimadzu, Kyoto, Japan). The prepared MLTs were placed between two plates and compressed on the tablet at a speed of 1 mm/min, causing fracture or delamination of the MLTs. The peak force obtained from the force–displacement plots was determined as the compaction force required to break the tablets.

### 3.7. Interfacial Strength of MF/EG-Loaded MLTs

The interfacial tensile strength of MF/EG-loaded MLTs was determined using a universal testing machine (JP/AG-50kNX; Shimadzu, Kyoto, Japan) as previously reported [26]. A newly designed holder was used to measure the convex-shaped tablets (Figure 3A). The MLTs were manually attached between the holders using super glue and hardened for 15 min. Subsequently, the upper holder was lifted at a speed of 1 mm/min to delaminate the tablets. The tablet interfacial strength (*σ*) was calculated as the maximum axial interfacial tensile force (*F*) divided by the cross-sectional area (*A*) of the tablet using Equation (2).

(2) $$\sigma =\frac{F}{A}$$

### 3.8. Determination of Surface Topography of MF/EG-Loaded MLTs

The surface roughness of the first layer following pre-compression was profiled using stylus profilometry (DektakXT, Bruker, Mannheim, Germany). A diamond-tip stylus (radius of 2 μm) was drawn along the first layer of the bilayer tablet with a movement speed of 285 µm/s and a tracking force of 1 mg. The scan size and temperature were set to 2000 µm and 25 °C, respectively. In total, 2100 points were scanned per tablet and the degree of surface roughness was characterized in accordance with International Standards Organization 4287 [46] as follows: Rz (defined as the sum of the height of the largest profile peak and the depth of the largest profile valley within a sampling length), Rp (defined as the maximum profile peak height within a sampling length), Rq (defined as the root mean square deviation from the mean line within a sampling length), and Ra (defined as the arithmetical mean deviation from the mean line within a sampling length).

The average maximum height of the profile Rz (Equation (3)) is the sum of the maximum profile peak height and the maximum profile valley depth for one sampling length.

(3) Rz=Rp+Rv

where *Rp* is largest profile peak height and *Rv* is the largest profile valley depth in one sampling length.

The root mean square roughness, *Rq* (Equation (4)), is the root mean square average of the roughness profile ordinates.

(4) $$Rq=\frac{1}{l}\int_{0}^{l}Z^{2}(x)dx$$

The average roughness *Ra* (Equation (5)) is the arithmetic average of the absolute values of the roughness profile ordinates.

(5) $$Ra=\frac{1}{l}\int_{0}^{l}\left|Z\left(x\right)\right|dx$$

where *l* is the scan length of the sample and *Z*(*x*) represents the height of the profile at each position *x*.

### 3.9. EDS-Equipped SEM Observations of Interfaces of Bilayer Tablets

The roughness of the first layer after pre-compression was examined using SEM (MIRA3 LMH, TESCAN, Czech Republic). The pre-compressed MF layer was mounted onto a copper stub with the surface facing up using double-sided carbon tape (Sungho Sigma, Suwon, Republic of Korea). The samples were then coated with a thin layer of Pt. Microphotographs of the coated samples were obtained using scanning electron microscopy at an acceleration voltage of 15 kV.

An EDS (Xflash4010, Bruker, Germany) equipped with a scanning electron microscope was further employed to track chloride (Cl) originating from MF at the interface and adjacent layers. After the pre-compression and subsequent main compression processes, the interface between the MF and EG layers was cross-sectioned using a razor. Samples were then mounted on a stud using double-sided electrical carbon tape. The energy resolution of the Si drift detector was set to 125 eV.

### 3.10. Statistical Analysis

Each experiment was performed at least three times, and the data are presented as the means ± standard deviations (SD). The linearity between the data was analyzed using Origin software (version 9.0, OriginLab Corporation, MA, USA). 

## 4. Conclusions

The effects of the PRE-P and the MAIN-P on the interfacial roughness, compaction breaking force, and interfacial strength of convex-shaped EG and MF MLTs were successfully evaluated using a profilometer and EDS-equipped SEM. A roughness analysis using a profilometer revealed that increasing the PRE-P led to a decrease in the surface roughness of the first layer, thus weakening the intermolecular adherence between the adjacent layers and decreasing the interfacial strength required to delaminate the tablets. Accordingly, using EDS-SEM, the inter-penetration at the interface was diminished when the roughness of the first layer was decreased in MF/EG MLTs. Therefore, we suggest that observing the surface roughness of the first layer using a profilometer and EDS-SEM can be a simple and effective tool to understand the delamination phenomenon of MLTs.

## Figures and Tables

**Table 1:** Composition of mixtures subjected to granulation processes.

	Function	Ingredient	Content (mg)	
			Bilayer Tablet	Trilayer Tablet
Third Layer	Active substance	MF	-	500
	Binder	PVP K30		15
	Lubricant	Magnesium stearate		2.5
	Controlled release excipient	Carbomer 934P		17.5
	Controlled release excipient	HPMC2208		65
	Controlled release excipient	Methacrylate copolymer		30
Second layer	Active substance	EG	6.8	6.8
	Diluent	Pregelatinized starch	9.0	9.0
	Diluent	Mannitol	71.1	71.1
	Disintegrant	Ac-Di-Sol	13.5	13.5
	Disintegrant	L-HPC	9	9
	Glidant	Colloidal silicon dioxide	1.3	1.3
	Lubricant	Magnesium stearate	3.1	3.1
	Colorant	Iron oxide	0.3	0.3
	Binder	HPC	2.7	2.7
First layer	Active substance	MF	1000	500
	Binder	PVP K30	30	15
	Lubricant	Magnesium stearate	5.0	2.5
	Controlled release excipient	Carbomer 934P	35	17.5
	Controlled release excipient	HPMC2208	130	65
	Controlled release excipient	Methacrylate copolymer	60	30

Abbreviations: EG, evogliptin tartrate; HPC, hydroxypropyl cellulose; L-HPC, low-substituted hydroxypropyl cellulose; HPMC, hydroxymethyl cellulose; MF, metformin HCl; PVP, polyvinylpyrrolidone.

**Table 2:** Physical properties of MF and EG granules prepared by the wet granulation method.

	EG Granules	MF Granules
LOD (%) ^1^	0.70 ± 0.09	0.74 ± 0.09
BD (g/mL) ^1^	0.46 ± 0.01	0.43 ± 0.01
TD (g/mL) ^1^	0.51 ± 0.01	0.48 ± 0.01
HR ^1,2^	1.11 ± 0.02	1.12 ± 0.02
CI (%) ^1,3^	9.79 ± 1.89	10.48 ± 1.90
Particle size d_0.1_ (μm) ^1,4^	16.18 ± 0.22	10.73 ± 0.25
Particle size d_0.5_ (μm) ^1,5^	38.33 ±1.86	44.74 ± 2.19
Particle size d_0.9_ (μm) ^1,6^	83.75 ± 2.74	110.93 ± 4.23
SPAN ^1,7^	1.76 ± 0.02	2.24 ± 0.02

Abbreviations: BD, bulk density; CI, Carr’s index; HR, Hausner’s ratio; LOD, loss on drying; TD, tap density. ^1^ Data represent means ± SD (*n* = 3). ^2^ calculated by dividing the tapped density by the bulk density; ^3^ calculated by dividing the difference between tapped density and bulk density by tapped density; ^4^ indicates the volume weighted diameters below which 10% of the total particles lie; ^5^ indicates the volume weighted diameters below which 50% of the total particles lie; ^6^ indicates the volume weighted diameters below which 90% of the total particles lie; ^7^ calculated by dividing the difference between d_0_._9_ and d_0_._1_ by d_0_._5_.

## Data Availability

Data is contained within the article.
